# Understanding the implementation of maternity waiting homes in low- and middle-income countries: a qualitative thematic synthesis

**DOI:** 10.1186/s12884-017-1444-z

**Published:** 2017-08-31

**Authors:** Loveday Penn-Kekana, Shreya Pereira, Julia Hussein, Hannah Bontogon, Matthew Chersich, Stephen Munjanja, Anayda Portela

**Affiliations:** 1 School of Public Health, Faculty of Health Sciences, Centre for Health Policy/MRC Health Policy Research Group, Private Bag X3, University of the Witwatersrand, Johannesburg, 2050 Gauteng South Africa; 2 Department of Infectious Disease Epidemiology, London School Hygiene and Tropical Medicine, Keppel Street, London, WC1E 7HT UK; 3 Department of Global Health and Development, London School Hygiene and Tropical Medicine, 15-17 Tavistock Place, London, WC1H 9SH UK; 4 Immpact, Institute of Applied Health Sciences, University of Aberdeen, Foresterhill, Aberdeen, AB25 2ZD Scotland; 5 Department of Maternal, Newborn, Child, Adolescent Health, World Health Organization, 20, Avenue Appia, 1211 Geneva, Switzerland; 6 Wits Reproductive Health and HIV Institute, Faculty of Health Sciences, University of the Witwatersrand, Johannesburg, South Africa; 7 Department of Obstetrics and Gynaecology, College of Health Sciences, University of Zimbabwe, Mazowe Street, Harare, Zimbabwe

**Keywords:** Maternity waiting homes, Shelters, Obstetric complications, Childbirth, Referral system, Low and middle-income countries

## Abstract

**Background:**

Maternity waiting homes (MWHs) are accommodations located near a health facility where women can stay towards the end of pregnancy and/or after birth to enable timely access to essential childbirth care or care for complications. Although MWHs have been implemented for over four decades, different operational models exist. This secondary thematic +analysis explores factors related to their implementation.

**Methods:**

A qualitative thematic analysis was conducted using 29 studies across 17 countries. The papers were identified through an existing Cochrane review and a mapping of the maternal health literature. The Supporting the Use of Research Evidence framework (SURE) guided the thematic analysis to explore the perceptions of various stakeholders and barriers and facilitators for implementation. The influence of contextual factors, the design of the MWHs, and the conditions under which they operated were examined.

**Results:**

Key problems of MWH implementation included challenges in MWH maintenance and utilization by pregnant women. Poor utilization was due to lack of knowledge and acceptance of the MWH among women and communities, long distances to reach the MWH, and culturally inappropriate care. Poor MWH structures were identified by almost all studies as a major barrier, and included poor toilets and kitchens, and a lack of space for family and companions. Facilitators included reduced or removal of costs associated with using a MWH, community involvement in the design and upkeep of the MWHs, activities to raise awareness and acceptance among family and community members, and integrating culturally-appropriate practices into the provision of maternal and newborn care at the MWHs and the health facilities to which they are linked.

**Conclusion:**

MWHs should not be designed as an isolated intervention but using a health systems perspective, taking account of women and community perspectives, the quality of the MWH structure and the care provided at the health facility. Careful tailoring of the MWH to women’s accommodation, social and dietary needs; low direct and indirect costs; and a functioning health system are key considerations when implementing MWH. Improved and harmonized documentation of implementation experiences would provide a better understanding of the factors that impact on successful implementation.

## Background

Ensuring births with a skilled attendant and births in health facilities has been the key focus of attempts to reduce maternal mortality in the last two decades [1, 2]. Many women in developing countries live far away or across difficult terrain from facilities. Transport is not always available, or may be difficult or too slow, particularly for women in labour, or when complications have developed [2]. Strategies typically designed for inaccessible areas aim to facilitate the timely movement of women from home to health facility by diminishing barriers that inhibit access to care such as distance, geography, seasonal barriers or the time of day. The interventions relate to improving infrastructure or transport, addressing the cost of transport or enabling communication between referral points [3]. One intervention designed to address accessibility are maternity waiting homes (MWHs). Maternity waiting homes are defined as lodgings or accommodation close to a health facility where women can stay before and sometimes after they give birth. Women staying in MWHs are then able to easily access services for essential childbirth care or obstetric or newborn complications at the nearby facility [4].

MWHs have been advocated for and implemented for over four decades [5]. Current maternal health strategies embrace MWHs, including the Campaign on Accelerated Reduction of Maternal, Newborn & Child Mortality in Africa (CARMMA) programme in South Africa [6], Saving Mothers Giving Lives in Zambia and Uganda [7], Gates funded projects in Malawi [8]and the Plan of Action to Accelerate the Reduction of Maternal Morality and Severe Maternal Morbidity for the Americas [9]. Different operational models of MWHs exist. In the past, MWH programmes targeted women most at risk of developing obstetric complications [10–14]. More recently, the focus has expanded to all pregnant women who would otherwise have problems accessing facilities for birth [3, 10, 15–17].

In 2015 the World Health Organization (WHO) published *Recommendations on Health Promotion Interventions for Maternal and Newborn Health* [4]. An intervention assessed within this guideline include MWHs. The Guideline Development Group reviewed the evidence collected and concluded that “MWHs are recommended to be established close to a health facility where essential childbirth care and/or care for obstetric and newborn complications is provided to increase access to skilled are at birth for populations living in remote areas or with limited access to services” p.5.

In addition to commissioning a systematic review to determine the evidence of effectiveness of MWHs on key maternal health outcomes, the WHO also commissioned a background document to analyse the context and conditions and factors that affect implementation of MWHs. This article builds on that background document. The objective of this paper is to share with policy makers and implementers who are thinking about implementing MWHs key learnings from other implementation experiences, so that they can apply lessons to their own contexts.

## Methods

This article is a secondary thematic analysis of studies identified in a systematic review of MWHs commissioned by WHO whose findings are summarized in the above-mentioned guidelines: [4] four existing systematic reviews [3, 18–20] and a systematic mapping of maternal health literature published from 2000 to 2012 were identified [21].

For this paper we included 29 studies identified through the above systematic reviews: 14 of these were included in the WHO-commissioned review and an additional 15 papers which were not included in the WHO-commissioned evidence review but included here as they described the implementation of MWHs, through qualitative or quantitative studies. The characteristics of the 29 studies included in this analysis are listed in Table 1.

**Table 1:** General characteristics of included studies

Title	Study Design	Setting	Scale^2^	Description of Intervention
Ande-michael et al. (2009)	Hospital-based before and after study with qualitative component	Eritrea, remote areas of two coastal regions of the Red sea	655,279 people 11 MWH	11 health facilities with MWH for women living at least 10 km distance from facility. MWHs had an ambulance for referal to higher level facilities for complications. During admission, consumables were provided to women. Community support provided through supplies. Equity considerations made for women residing more than 10 km from health facility. Staff at MWH were trained. Part of a strategy implemented by MOH
Chandra-mohan et al. (1994)	Hospital cohort (childbirth outcomes over time)	Zimbabwe, Rural	208,000 people 1 MWH	Free self-catering temporary accommodations 150 m from labour ward. Women advised to stay at MWH from 36 weeks gestation. Target population was women identified as risk in ANC. MWH offered ANC and health education.
Chandra-mohan et al. (1995)	Cohort analytic (two group pre + post)	Zimbabwe, Rural	208,000 people 1 MWH	See Chandramohan et al., 1994
Danel et al. (2003)	World bank report	Honduras, National	Population nr 5 MWH	Attached to rural hospitals.
Ecker-mann et al. (2008)	Case study with qualitative components	Lao People’s Democratic Republic (PDR), Remote-rural	27,539 people No MWH, 17 to be built	Improve maternal outcomes in remote communities with a high proportion of ethnic minorities and disadvantaged groups economically and in health indices. Women provided with nutrition and baby care training, handicraft training and have opportunity to earn an income while staying at MWH. All given information and opportunities for micro-credit initiatives. MWHs designed for privacy before, during and after birth (for uncomplicated births conducted in MWH in traditional birthing position)
Feresu et al. (2003)	World bank report	Zimbabwe National	Overview of 255 MWH	
Fraser (2008)	Case study	Peru, Rural and urban	Population nr 2 MWH (390 available nationally)	Reported outcomes of key interventions to address MMR in Peru. MWH near health centres that refer cases to hospitals. MWH are part of a strategy implemented by MOH
Garcia Prado et al. (2012)	Cross-sectional survey and qualitative components	Nicaragua, Rural	Population nr 18 MWH	Women spend 2 weeks before and 1 week after childbirth at MWH, where food and lodging is provided. Most homes extend their services beyond medical visits and education on SHR, offering advice and counselling on diverse issues (domestic violence, selling handmade prouducts, and obtaining identify cards or land titles). Women referred from mobile health teams and TBAs. Situated near health centres. MOH has a strategy to promote MWHs.
Gaym et al. (2012)	Hospital based cohort with a qualitative component	Ethiopia, Rural	Population nr 9 MWH	Faith based organizations pioneered the construction of MWHs in Ethiopia since the late 1980’s, then adopted by NGOs as well as public health facilities. Conditions within each varied, activities included outreach to increase community awareness of MWHs. Women referred by staff at peripheral health facilities, and outreach teams. Women also came based on recommendations from other women who had used facility. Situated within compound of health facility.
Gorry (2011)	Case study	Cuba, Rural and urban	Population nr 327 MWH	15 MWHs were introduced in 1962 and grew to 327. Existing houses are reconditioned to create a home-like environment for monitoring health and wellbeing of woman and fetus. Concept has been further developed to emphasize nutrition and diet, and provision of ambulatory services so women can take meals and classes at MWH, but return home in the evenings. MWHs follow guidelines designed by Ministry of Public Health’s maternal child health program in collaboration with UNICEF, describing criteria for admission, diagnostic and clinical guidelines for identifying risk factors and protocols for treatment in MWHs.
Kelly et al. (2010)	Hospital cohort (childbirth outcomes over time)	Ethiopia, Rural	800,000 people 1 MWH	40 bed MWH, located within hospital grounds. Original facility built in 1973 in local style with thatched roof, which caught fire in 1999; replaced by corrugated roof. A companion resides at MWH, finds firewood and food, and cooks for her. High-risk women spend last few weeks of pregnancy in MWH.
Knowles et al. (1988)	Case study	Malawi	Population nr 1 MWH	Women referred from other medical facilities and can self-refer. Situated in hospital ground.
Larsen et al. (1978)	Hospital cross sectional survey	South Africa, Rural	nr	Nr
Lori et al. (2013a)	Qualitative study	Liberia, Rural, post conflict	78,446 people 5 MWH	Served women affected by conflict. Women self-refer. Situated near health facilities.
Lori et al. (2013b)	Cohort analytic (two group pre + post)	Liberia, Rural post conflict	>50,000 people 4 MWH	Served women affected by conflict.
Martey et al. (1995)		Ghana, Rural	131,229 people 5 MWH	Nr
Millard et al. (1991)	Hospital cohort study	Zimbabwe, Rural	Population nr 1 MWH	Women self-referred themselves to the facility. 2 min walk from hospital. MOH policy exists supporting MWHs.
Mramba et al. (2010)	Cross sectional survey, qualitative components	Kenya	Population nr 1 MWH	50 m from the maternity unit at a District Hospital. It has a capacity of 40 people: 20 pregnant women and 20 healthcare workers. Referrals mostly by health workers. Referrals from health workers.
Poovan et al. (1990)	Hospital cross-sectional survey	Ethiopia, Rural	300,000 people 1 MWH	Women referred during outreach ANC conducted by nurse midwives and TBAs. Situated close to the hospital.
Ruiz et al. (2013)	Qualitative study	Guatemala, Urban	Population nr 2 MWH	Focus on attracting indigenous women. Women referred from TBAs and health centre physicians. Women could also self-refer. 3 km from the hospital. Part of a MOH strategy to increase utilisation in this region.
Schooley et al. (2009)	Qualitative inquiry (focus groups and in-depth key informant interviews, unstructured, focused observations)	Guatemala	Population nr 1 MWH	Focus on increasing utilisation of health services by indigenous women. Situated adjacent to a local hospital.
Shrestha et al. (2007)	Cross-sectional survey and qualitative component	Nepal, Lowland conflict	Population nr Study not linked to existing MWH (27 MWH available)	Working in a context of conflict. MOH supported MWH to increase health facility utilisation.
Spaans et al. (1998)	Household-level cross-section	Zimbabwe	Population nr 4 MWH	In the hospital grounds.
Tumwine et al. (1996)	Cohort analytic (two group pre + post)	Zimbabwe	100,000 people Number of MWH nr	Women referred by health centre staff, TBAs and could refer themselves. 100 m from hospital.
van Lonkhuij-zen et al. (2003)	Hospital cross-section	Zambia, Rural	60,000 people 1 MWH	Women referred during monthly outreach clinics conducted by midwives. Situated next to hospital.
Wessel(1990)	Case study	Nicaragua, Rural	Population nr 1 MWH	Aimed at supporting refugees from the civil war. Self-referral.
Wild et al. (2012)	Interrupted time series	Timor-Leste Remote-rural	>100,000 people 2 MWH	Connected by a walkway to the hospital, and near a health centre. MOH run as part of their maternal health strategy.
Wilson et al. (1997)	Qualitative study, with MWH utilisation rates	Ghana, Rural	126,000 people 1 MWH	Referrals from private midwives and health posts. Situated in an unused ward in the hospital.

1 Year of study or report; 2 Catchment population reportedly covered by MWH and number of MWH included in article; 3 Health indices reported as background levels in the article only, pertinent to locality, population of interest and time period where available. Health indices as a result of the MWH intervention not included

Abbreviations: MMR = maternal mortality ratio/100000, PMR = perinatal mortality/1000, SBA = skilled birth attendance, IDR = institutional delivery rate, HB = home births, ANC = antenatal care, PHC = primary health centres, TBA = traditional birth attendants, MOH = ministry of health nr = not reported

We used the Supporting the Use of Research Evidence framework (SURE) framework [22] to identify different contextual and health system factors that affect implementation of MWHs and conducted data extraction on the key themes (See Table 2). The relevant information extracted on perspectives of women who used MWHs, community stakeholders, health care providers and other stakeholders; health service delivery factors; and social and political factors is presented in Table 3 and summarized below.

**Table 2:** Guide for extracting data and emergent themes

Content of interest	Themes which emerged
• Demographic, socio-cultural, economic, country context • Health indicators • Health system characteristics • Policy • Community characteristics	General characteristics of context and MWH
• Timeline	
• General equity considerations (e.g. gender, ethnic, racial, marginalized and vulnerable populations)	
• Assumptions, theory of change, models or frameworks used to guide program design and implementation	Definition or description of MWH, and hypothesis or reasoning for establishment of MWH
• Program context (key actors, organizations, participants, implementing partners, & who did what, who initiated the program)	Administrative set-up and maintenance of MWH
• Monitoring and evaluation system characteristics	
• Cost of intervention, financial considerations (e.g. incentives, compensation), source of funding	
• Structural and financial support and considerations (organizational systems, training/education and support for implementers/actors/participants)	
• Description of approach/intervention (process used)	Description of physical facilities, utilities provided and infrastructure of MWH (e.g. bed size, number of rooms, cooking, sanitation facilities)
	Health related activities at the MWH (e.g. health education, training, antenatal care, income generation)
• Inhibiting factors, challenges and enhancing factors	Barriers and enabling factors related to MWH based on perceptions of (a) community (b) health workers (c) authors of articles
• Sustainability

**Table 3:** Barriers and enablers to implementation of MWHs analysed using the SURE framework

Level	Barriers	Article	Enablers	Article
Main Stakeholders from the Community-women & families	*Knowledge and Skills*			
	Lack of knowledge of MWH	- Mramba et al. 2010 - Ruiz et al. 2013 - Shrestha et al. 2007	Awareness of MWHs and services offered through community outreach and mobilization is high among women	- Garcia Prado et al. 2012 - **Gaym et al. 2012** - **Kelly et al. 2010** - **Poovan et al. 1990** - Schooley et al. 2009 - **Wild et al. 2012**
	Women do not remember the date of their last period, so unsure about expected due date-point of entry into MWH and duration of stay uncertain and may be prolonged	- Eckermann et al. 2008 - **Spaans et al. 1998**		
	*Attitudes regarding programme acceptability, appropriateness and credibility*			
	Traditional childbirth practices not accommodated	- Eckermann et al. 2008 - Ruiz et al. 2013	Integration of cultural norms and expectations into the care provided at the MWH and associated health facility	- Fraser 2008 - Lori et al. 2013b - Ruiz et al. 2013 - Schooley et al. 2009
	Family members (husbands and mothers in law) don’t allow women to use MWHs and no one left at home to do household chores or provide child care	- Mramba et al. 2010 - Garcia Prado et al. 2012 - Lori et al. 2013b - Ruiz et al. 2013 - Nhindiri et al. 1996	High awareness of benefits and acceptability of MWHs and facility birth among family and community members. Family and community actively involved through educational outreach and involved in decision-making.	- Garcia Prado et al. 2012 - **Gaym et al. 2012** - **Kelly et al. 2010** - Lori et al. 2013a - Ruiz et al. 2013 - Schooley et al. 2009 - **Wild et al. 2012**
			High acceptability of facility births and use of MWHs among women	- **Gaym et al. 2012** - **Kelly et al. 2010**
	Health workers and users of MWH have different ethnicities which result in communication problems	- Ruiz et al. 2013		
	Companion not allowed or unable to accompany	- Eckermann et al. 2008 - **Gaym et al. 2012** - Ruiz et al. 2013 - Schooley et al. 2009 - **Wild et al. 2012**		
Healthcare Providers Involved in Implementing MWH	*Knowledge and Skills*			
	Without access to technologies, not possible for health workers to predict date of delivery so duration of stay is uncertain and prolonged stay at MWH might occur	- Eckermann et al. 2008		
	*Attitudes regarding programme acceptability, appropriateness and credibility*			
	Health workers and users of MWH have different ethnicities which result in communication problems	- Ruiz et al. 2013		
Other Stakeholders	*Knowledge and Skills*			
			Training TBAs and integrating TBAs into the birthing process helped encourage women to use MWHs and deliver in facilities	- **Andemichael et al. 2009** - Lori et al. 2013a - Lori et al. 2013b - **Poovan et al. 1990** - Schooley et al. 2009
Health Service Delivery Factors	*Accessibility of care*			
	Geographical - MWH is too far	- Schooley et al. 2009 - Shrestha et al. 2007	MWH located close to the hospital	- Nhindiri et al. 1996
	Cost - MWH users incur costs for travel - MWH users incur costs of staying at facility - MWH use leads to costs of subsequent delivery in health facility	- Eckermann et al. 2008 - Garcia Prado et al. 2012 - **Gaym et al. 2012** - Ruiz et al. 2013 - Schooley et al. 2009 - Spaans et al. 1998 - Wessel et al. 1990 - Wilson et al. 1997	Removal/reduction of costs associated with using the MWH and/or subsequent institutional delivery	- Kelly et al. 2010 - Ruiz et al. 2013 - Spaans et al. 1998 - Wessel 1990
Health Service Delivery Factors	*Training*			
	No regular visits by health workers or link to obstetric care are insufficient and unclear	- Wilson et al. 1997 - Mramba et al. 2010 - Gaym et al. 2012	Daily visits to the MWH by midwives	- **Poovan et al. 1990**
			Intensive training of health providers in MWH and facilities to provide good quality of care	- Fraser 2008 - Gorry 2011
	*Communication*			
	No clear communication of what to expect at the MWH while at the MWH	- Mramba et al. 2010		
	Health workers attitudes are not good	- Garcia Prado et al. 2012 - Lori et al. 2013b - Wilson et al. 1997		
	*Information Systems*			
	No registration and linkage of MWH records with health information system	- Danel et al. 2003		
			Strong referral and communication systems between MWH and associated facilities, including transportation and communication equipment	- **Chandramohan et al. 1995** - Gaym et al. 2012 - **van Lonkhuij-zen et al. 2003**
	*Facilities*			
	- Lack of privacy in MWH - No space for relatives or companions to stay - Poor toilet and bathing facilities - Kitchen facilities are poor or inadequate - Food not provided leading to differential access to food for MWH users. Women had to travel back to their homes to replenish supplies - A lack of space for postpartum women	- Eckermann et al. 2008 - **Gaym et al. 2012** - **Kelly et al. 2010** - Lori et al. 2013b - Mramba et al. 2010 - Nhindiri et al. 1996 - Ruiz et al. 2013- Schooley et al. 2009 - Shrestha et al. 2007 - **Wild et al. 2012** - Wilson et al. 1997	MWH provides and maintains all needed facilities, including basic infrastructure such as electricity, kitchen/food facilities, and bathing and toilets. MWH also provided a space for companions and family members to stay with the pregnant woman.	- Lori et al. 2013a - Mramba et al. 2010 - Nhindiri et al. 1996 - Ruiz et al. 2013
	Useful activities to occupy women’s time and provide knowledge and skills are not organised or insufficient (for example: entertainment, income generation skills, health education)	- Eckermann et al. 2008 - Ruiz et al. 2013 - Mramba et al. 2012 - Lori et al. 2013b	Activities to occupy women’s time, including education and income generation activities, helped improve acceptability and use of MWH among women	- Gorry 2011 - Ruiz et al. 2013 - **Tumwine et al. 1996** - Wessel 1990
	*Intervention integrity*			
			Comprehensive provision of good quality care, across the continuum of care, in both the MWH and health facilities associated with the MWH	- Gorry 2011 - **Poovan et al. 1990** - Schooley et al. 2009 - **Tumwine et al. 1996**
Social and Political Factors	*Legislation or regulations*			
			Enabling policy environment, which included inclusion of supportive MWH policies in national and/or local legislation	- Fraser 2008 - Gorry 2011 - Lori et al. 2013b - Millard et al. 1991
	*Sustainability*			
	Lack of community involvement in MWH set up, support and maintenance	- **Poovan et al. 1990** - **Kelly et al. 2010** - Lori et al. 2013a - Ruiz et al. 2013 - Shrestha et al. 2007 - Wilson et al. 1997	Involve community members and family in the design, development, and maintenance of the MWH	- Lori et al. 2013b - **Poovan et al. 1990** - Schooley et al. 2009
		-	MWH and facilities are able to adapt to changing health needs of women. For example, in Cuba, an economic crisis meant needing to focus and integrate nutrition improvement for pregnant women in the MWH	- Gorry 2011

Articles that are highlighted in bold are those that were included in the systematic review of effectiveness of MWHs

## Results

Table 1 gives information on study design of the papers included. Fourteen of the papers included were impact studies, including 11 cohort studies, two cross-sectional studies and one review of records. The other fifteen papers were either qualitative or mixed method in research design. In two cases, no research design was reported. The dates of the studies ranged from 1978 to 2013, with the majority published between 2003 and 2013. Below we organize the analysis of implementation factors extracted from the different studies into five main categories.

### Maternity waiting homes settings and target populations

The included studies on MWHs were from countries in Africa (nine countries – Eritrea, Ethiopia, Ghana, Kenya, Liberia, Malawi, South Africa, Zambia, Zimbabwe), Latin America (four countries – Cuba, Guatemala, Honduras, Nicaragua, Peru], and Asia (three countries - Lao PDR, Nepal and Timor-Leste).

Reported interventions were generally confined to a few districts involving one to five MWHs. However, articles from Cuba and Peru reported larger numbers of MWHs being built [16, 23]. The majority of settings were rural. Some specifically targeted conflict areas, indigenous women, the socially excluded, or poor people. [17, 24, 25] Depending on the location of the MWH, women travelled from less than 5 km to 400 km to reach the closest MWH [10, 15]. Along with large distances, several studies reported women having to cross difficult terrain to reach the facility. Most MWHs were situated next to a hospital facility, which provided essential childbirth care services and care for complications (comprehensive obstetric care services), although a few were placed near health centres that provided only essential childbirth care. Practices of referral to MWHs varied; women were referred by health professionals, from antenatal clinics or self-referred.

### Administrative set up and maintenance of maternity waiting homes

There is diversity in the stakeholders who took responsibility to establish the MWHs in the different studies included. The programmes in Cuba and Peru were large scale and, at least initially, adequately funded and supported by their respective National Ministries of Health. These MWHs were implemented as part of a national programme to improve maternal health outcomes, alongside new protocols, staff training, and improved referral and support for women [16, 23]. Aside from these examples, little information was found on policy support for MWHs at a national level.

The remainder of the MWHs consisted of isolated projects, supported by non-governmental and donor organisations. A number of articles reported community support and contribution to the setup and ongoing running of the MWH. The need for the community to be involved in the set up and maintenance of the MWH was identified in three studies, and six studies identified the absence of community involvement as a reason for low utilization of the MWH programme [13, 17, 26–29].

Several studies reported on MWH residents incurring user fees for antenatal care or childbirth services [10, 17, 25, 29–33]. The removal or reduction of costs associated with using the MWH and subsequent institutional birth were noted as important strategies for increasing MHW use. In two studies, financial incentives were even offered for women, who were charged less for childbirth services if they stayed in the MWH [13, 17]. The provision of free food by the MWH varied across settings. In Cuba, meals were provided and tailored to the nutritional needs of each woman in consultation with dieticians at the MWH [16], while in other MWHs, food or kitchen facilities were available for the women to arrange their own meals [10, 16, 18, 31, 33]. However, in instances where women and their families were required to provide their own meals, inequalities in terms of volume and quality of food emerged among the women [10, 13, 33, 34].

A number of studies reported that simply building a MWH did not overcome barriers to accessing care as women still needed financial resources to get to the MWH [15, 17, 25, 28, 30]. The cost of public transportation to reach the MWH was a common barrier to its use and varied depending on the mode of transport distance and time of day [13]. Considerable costs were also reported for securing private transport. The comfort and speed of the transport, as well as the terrain covered were other elements considered by women [13, 30]. In Laos PDR, women were refunded transport costs. In Nicaragua and Laos PDR, women and their families indicated that upfront support for transportation costs would be important [23, 30].

### Physical infrastructure and facilities provided

A range of building types were used for MWHs, including unused wards of hospitals [29], traditional huts [12] and purpose-built structures. Some buildings had several separate rooms, each with a few beds [27], while others had large dormitories [17]. Total bed space ranged from 4 to to 83 [31]. In planning for the construction of a MWH in South Africa, Larsen et al. estimated that the size of a MWH should be based on 500 women per 1000 births in a district, with each stay averaging two weeks [35].

Living and social spaces, as well as utilities like electricity or water, kitchens, cooking utensils, toilets and bathrooms, lockers, bedding and firewood, were described in some papers. From the perspectives of women who used the facility, a lack of privacy, poor toilet and bathing facilities, poor or inadequate kitchen facilities, the non provision of food, and lack of space for women to stay post-partum were considerable barriers to MHW use. [10, 13, 15, 17, 25, 27–30, 36, 37] Overall, MWHs were better used and accepted by women and their families when they provided basic infrastructure and facilities such as those mentioned above [17, 27, 36, 37]. In one MWH in Ethiopia the availability of a hot shower was very popular with women [13].

In some situations, accommodation was provided for relatives, including mother in laws [17, 28]. Women cited companions not being allowed – either at the MWH or in the facility – as an additional factor undermining acceptability of MWHs [10, 15, 17, 25, 30]. Finally, in interviews with women and families, acceptability of MWH was noted as being higher if activities were available for women to do while awaiting childbirth, such as health education and income generation activities [14, 16, 17, 33].

### Health services and linkages with the facility

Various criteria were used to accept women into MWHs, from identified obstetric risk factors for complications, to open admission. Women were advised to stay for between one to four weeks before childbirth and, in some MWHs, for up to seven days after birth. Two studies suggested that sometimes uncertainty around a woman’s due date meant that she did not know when it was appropriate to come to the MWH [30, 32]. MWHs were sometimes also used as places for women to stay before and after undergoing postpartum tubal ligation at the hospital or other health facilities [10, 33, 38].

Studies suggest that strong referral and communication systems between the MWH and the facilities they are linked to are important, as well as a focus on providing high quality care in both the MWH and the facility linked to the MWH [14, 16, 17, 25, 26, 32, 34, 37, 39, 40]. The type and quality of maternity care services received by women varied. Three studies noted that there were no regular visits by health care providers to the MWH and that referral from the MWH to the facility was not smooth [10, 29, 36]. In other sites, women regularly attended the nearby health facility, or were visited in the MWH by staff from the facility [14, 26, 31]. Standard guidelines for care processes, including criteria for admissions, diagnostic and clinical guidelines for identifying risk factors, and protocols for treatment in MWH settings, were reported in Cuba [16].

### Community involvement and sensitivity to cultural norms

Linking with traditional birth attendants (TBAs) was seen as enabling the success of MWH programmes. Five studies identified this as critical to facilitating access to MWHs, specifically, through the training of TBAs and the integration of them into the preparation for birth and birthing process both at the MWH and at the facility [24–27, 39].

In four studies, the integration of cultural norms around birthing and improved awareness that the MWH provided respectful and humanized care were key to getting women and their families to use both MWH and the nearby facility for birth [17, 23–25]. Finally, on the issue of cultural norms, concerns were expressed by women in Guatemala around health workers belonging to a different cultural group to those attending MWH, and the potential for this to pose linguistic challenges and also to undermine respect for a woman’s cultural beliefs [17, 31].

A number of studies identified outreach to the community, often using existing community health structures, as key to the success of a MWH project [25–28].

Community involvement was important to identify cultural factors that affected the use of the MHW; for example, family members, namely the husband or mother-in-law, would not “allow” women to use the MWH or to be away from the household for an extended length of time due to childcare and other household duties [17, 27, 31, 36, 37]. Awareness building efforts were especially important in places where community members had little knowledge of the MWH, which in itself constituted an important barrier to MWH use [17, 28, 36].

Overall, activities to increase community awareness of the MWH services were considered a vital facilitator of MWH uptake [10, 13, 15, 25, 26, 31]. MWHs were embraced in those communities where family members and the larger community had been made aware of the importance of facility births [10, 13, 15, 17, 24, 25, 28, 31].

## Discussion

### Limitations and research gaps

We identified several limitations in this review. The wide variations in the organization, functioning and operationalization of MWHs, and how women were screened for MWH residence means that the studies are difficult to compare.

Most papers did not specifically set out to either document the contextual factors or assess barriers and facilitators. A number of factors that may play a key role on the implementation of these programmes were not reported (see Table 1). Surprisingly, there was relatively little in the reviewed literature around health care workers attitudes towards MWHs and how these affected implementation. Issues such as community participation were stressed as important in many articles, but what was meant by community participation and how community participation was ensured was not expanded upon. Studies that did seek community input often obtained information from women who were already users of facilities, rather than non-users. Nonetheless the included studies provide rich findings.

The studies included were generally of small-scale projects, although examples of scaled-up MWHs were available from Peru and Cuba. We have only drawn from published literature, but we are aware that there may be other experiences of implementation of MWH programmes from which lessons could be drawn, as many programmes may not be implemented as part of research or with a research component. This is particularly true of programmes implemented by National Ministries of Health.

Publication bias cannot be ruled out although the collection of systematic reviews and extensive search strategy for the Mascot mapping aimed to minimise this [21].

When considering implementing MWHs, key factors to be addressed include: 1) Community engagement, ensuring input is obtained from women and other community members as to the design and running of the MWH, identification of barriers to the use of MWH’s that need to be addressed and recommendations about how the community can be involved in maintaining the quality of the MWH’s; 2) the quality of the MWH structure, including cleanliness, living conditions, and the safety of women staying there; 3) the quality of maternity care services provided at the corresponding health facility; and 4) the financial and operational sustainability of the MWH.. The literature suggests that there is not one model that fits the diversity of contexts but it is clear there are multiple issues that require discussion with key stakeholders in order to address the factors that will affect implementation and ensure integration in to the health system.

Maternity Waiting Homes are not isolated interventions and one of the key challenges to its successful implementation is how well it can be embedded within the health system and integrated within community patterns, preferences, behaviours and other related services. Some interventions have presumed the following: women find birth in a health facility acceptable; the financial and indirect costs of residing away from home are affordable; and women’s basic rights to comfort, companionship, tradition, information and quality of care are respected. However the studies in this review have shown that these elements are variable and heavily dependent on the local context. We also see that there are multiple factors that affect care seeking for childbirth services; distance is only one factor. The MWH needs to be imbedded in a programme that addresses the other factors including costs, household decision making, knowledge of services, women and community perceptions of quality of care, etc.

The community perspectives in the studies reviewed demonstrate there is general awareness of the benefits offered by MWH, particularly when the community is consulted and involved. Involvement can range from participation in a governing committee, to faith-based organizations spearheading the physical construction of MWH, to community volunteers supporting individuals or running programmes within the facility.

The literature also suggests that it is important that all those involved in promoting maternal and newborn health and providing maternity care services must know about MWHs. It is likely that provision of MWH should be included in community health workers training, birth and complication preparedness, voucher programmes and other efforts to improve the levels of birth with a skilled attendant in rural and remote areas.

The ‘readiness’ of the linked health facility is also crucial. The literature suggests that women will not use MWH even when they are available if they are not confident about the care they will receive in the linked health facility. The quality of care (both respectful and medical quality) provided in the health facility should be adequate to improve both acceptability and health outcomes of childbirth.

## Conclusion

At policy level, there may be utility in development of guidelines and protocols on the physical infrastructure, utilities and services provided at the MWH, and community consultation. Additionally, clear identification of capital costs is required, along with a functioning management structure, regular flow of resources for maintenance and a defined relationship with the linked health facility and the health system.

Improved and harmonized documentation of implementation experiences would provide a better understanding of the factors that impact on successful implementation. As is illustrated in Table 1 many of the articles did not record key information that would have been useful to enable implementation lessons to be learnt.

## Abbreviations

ANC: Antenatal Care
CARMMA: Campaign on Accelerated Reduction of Maternal, Newborn and Child Mortality in Africa
HB: Home birth
MMR: Maternal Mortality Ratio
MOH: Ministry of Health
MWH: Maternity Waiting Homes
NGO’s: Non-Governmental Organisations
SRH: Sexual and Reproductive Health
SURE: Supporting the Use of Research Evidence Framework
TBA: Traditional Birth Attendants
WHO: World Health Organization

## References

[1] Campbell OM, Graham WJ (2006). Strategies for reducing maternal mortality: getting on with what works. Lancet.

[2] WHO UNFPA UNICEF World Bank (1999). Reduction of Maternal Mortality: A Joint WHO/UNFPA/UNICEF/World Bank Statement.

[3] Holmes W, Kennedy E (2010). Reaching emergency obstetric care: overcoming the ‘second delay’.

[4] Organisation WH (2015). WHO Recommendations on Health Promotion Interventions for Maternal and Newborn Health.

[5] World Health Organisation. Maternity Waiting Homes: A review of experiences. Geneva: World Health Organisation, Maternal and Newborn Health Safe Motherhood Unit. Division of Reproductive Health; 1996.

[6] United Nations Population Fund. No Woman Should Die Giving Life 2015 27th October 2015. Available from: http://carmma.org/update/carmma-high-level-event-african-leaders-unite-“prioritise-women-and-childhealth”.

[7] Kruk ME, Galea S, Grepin KA (2013). External Evaluation of Saving Mothers, Giving Life.

[8] University of North Carolina. UNC to improve safe motherhood effort in Malawi2015 27th October 2015 Available from: http://news.unchealthcare.org/som-vital-signs/2013/july-18/with-an-8-million-grant-from-the-bill-and-melinda-gates-foundation-unc-will-improve-safe-motherhood-in-malawi.

[9] Latin American Center for Perinatology Women and Reproductive Health. Plan of action to accelerate the reduction of maternal mortality and severe maternal morbidity: monitoring and evaluation strategy. Montevideo: 2012.

[10] Gaym A, Pearson L, Soe KW (2012). Maternity waiting homes in Ethiopia--three decades experience. Ethiop Med J.

[11] Chandramohan D, Cutts F, Chandra R (1994). Effects of a maternity waiting home on adverse maternal outcomes and the validity of antenatal risk screening. Int J Gynaecol Obstet.

[12] Millard P, Bailey J, Hanson J (1991). Antenatal village stay and pregnancy outcome in rural Zimbabwe. Cent Afr J Med.

[13] Kelly J, Kohls E, Poovan P, Schiffer R, Redito A, Winter H (2010). The role of a maternity waiting area (MWA) in reducing maternal mortality and stillbirths in high-risk women in rural Ethiopia. BJOG.

[14] Tumwine JK, Dungare PS (1996). Maternity waiting shelters and pregnancy outcome: experience from a rural area in Zimbabwe. Ann Trop Paediatr.

[15] Wild K, Barclay L, Kelly P, Martins N (2012). The tyranny of distance: maternity waiting homes and access to birthing facilities in rural Timor-Leste. Bull World Health Organ.

[16] Gorry C (2011). Cuban maternity homes: a model to address at-risk pregnancy. MEDICC Rev.

[17] Ruiz MJ, van Dijk MG, Berdichevsky K, Munguia A, Burks C, Garcia SG (2013). Barriers to the use of maternity waiting homes in indigenous regions of Guatemala: a study of users' and community members' perceptions. Cult Health Sex.

[18] van Lonkhuijzen L, Stekelenburg J, van Roosmalen J (2012). Maternity waiting facilities for improving maternal and neonatal outcome in low-resource countries. Cochrane Database Syst Rev.

[19] Hussein J, Kanguru L, Astin M, Munjanja S (2012). The effectiveness of emergency obstetric referral interventions in developing country settings: a systematic review. PLoS Med.

[20] Lee AC, Lawn JE, Cousens S, Kumar V, Osrin D, Bhutta ZA (2009). Linking families and facilities for care at birth: what works to avert intrapartum-related deaths?. Int J Gynaecol Obstet.

[21] Chersich MBD, Dumbaugh M, Penn-Kekana L, Thwala S, Bijlmakers L, Vargas E, Kern E, Kavanagh J, Dhana A, Becerra-Posada F, Mlotshwa L, Becerril-Montekio V, Mannava P, Luchters S, Pham MD, Portela AG, Rees H (2016). Mapping of research on maternal health interventions in low- and middle-income countries: a review of 2292 publications between 2000 and 2012. Global Health.

[22] Evidence-Informed Policy Network. SURE Guides for Preparing & Using Evidence-Based Policy Briefs GENEVA: WHO; 2011 [27th October 2015]. Available from: http://www.who.int/evidence/sure/guides/en/.

[23] Fraser B (2008). Peru makes progress on maternal health. Lancet.

[24] Lori JR, Wadsworth AC, Munro ML, Rominski S (2013). Promoting access: the use of maternity waiting homes to achieve safe motherhood. Midwifery.

[25] Schooley J, Mundt C, Wagner P, Fullerton J, O'Donnell M (2009). Factors influencing health care-seeking behaviours among Mayan women in Guatemala. Midwifery.

[26] Poovan P, Kifle F, Kwast BE (1990). A maternity waiting home reduces obstetric catastrophes. World Health Forum.

[27] Lori JR, Munro ML, Rominski S, Williams G, Dahn BT, Boyd CJ (2013). Maternity waiting homes and traditional midwives in rural Liberia. Int J Gynaecol Obstet.

[28] SD S. Feasibility study on establishing Maternity Waiting Homes in remote areas of Nepal. Regional Health Forum 2007;11(2):33–38.

[29] Wilson JB, Collison AH, Richardson D, Kwofie G, Senah KA, Tinkorang EK (1997). The maternity waiting home concept: the Nsawam, Ghana experience. The Accra PMM Team. Int J Gynaecol Obstet.

[30] Eckermann E, Deodato G (2008). Maternity waiting homes in Southern Lao PDR: the unique 'silk home'. J Obstet Gynaecol Res.

[31] Garcia Prado A, Cortez R (2012). Maternity waiting homes and institutional birth in Nicaragua: policy options and strategic implications. Int J Health Plann Manag.

[32] Spaans WA, van Roosmalen J, van Wiechen CM (1998). A maternity waiting home experience in Zimbabwe. Int J Gynaecol Obstet.

[33] Wessel L (1990). Casa Materna brings care to rural women in northern Nicaragua. Maternal mortality and morbidity: a call to women for action.

[34] Chandramohan D, Cutts F, Millard P (1995). The effect of stay in a maternity waiting home on perinatal mortality in rural Zimbabwe. J Trop Med Hyg.

[35] Larsen J, Muller E (1978). Obstetric care in a rural population. S Afr Med J.

[36] Mramba L, Nassir FA, Ondieki C, Kimanga D (2010). Reasons for low utilization of a maternity waiting home in rural Kenya. Int J Gynaecol Obstet.

[37] Pea N (1996). A community-based study on utilisation of maternity services in rural Zimbabwe. African Journal of Health Services.

[38] Danelwith IR, A, . Honduras, 1990–1997 In: Koblinsky M, editor. Reducing Maternal Mortality: learning from Bolivia, China, Egypt, Honduras, Indonesia, Jamaica and Zimbabwe. Health, nutrition, and population series. Washington, DC: The World Bank; 1993.

[39] Andemichael GH, Haile B, Kosia A, Mufunda J (2008). Journal of Eritrean Medical Association. Maternity Waiting Homes: A panacea for maternal/neonatal conundrums in Eritrea.

[40] Feresu SN, M Mumbwanda, L Zimbabwe, 1980–2000. In: Koblinsky M, editor. Reducing Maternal Mortality: learning from Bolivia, China, Egypt, Honduras, Indonesia, Jamaica and Zimbabwe. Health, nutrition, and population series. Washington DC: The World Bank; 2003.

